# Audiological alterations in resistance to thyroid hormone syndrome: emphasizing lifelong assessment

**DOI:** 10.20945/2359-4292-2024-0078

**Published:** 2025-01-31

**Authors:** Alexandre Machado Silva de Oliveira, Viviane Motti, Jessica Casemiro, Pedro Resende Rende, Caio Leônidas Oliveira de Andrade, Luciene da Cruz Fernande, Helton Estrela Ramos

**Affiliations:** 1 Faculdade de Medicina, Universidade Federal da Bahia, Salvador, BA, Brasil; 2 Departamento de Biorregulação, Instituto de Ciências da Saúde, Universidade Federal da Bahia, Salvador, BA, Brasil; 3 Curso de Fonoaudiologia, Universidade do Estado da Bahia, Salvador, BA, Brasil; 4 Instituto Multidisciplinar de Reabilitação e Saúde, Universidade Federal da Bahia, Salvador, BA, Brasil

## Abstract

The aim of this study was to investigate the long-term audiological consequences of resistance to thyroid hormone (RTH) syndrome. The cochlea and inner ear express thyroid hormone receptor beta (THRB) in developmental stages. Hearing loss is frequent in subjects with RTH syndrome; however, the long-term impact of insufficient thyroid hormone action in the auditory system remains unknown. Subjects with RTH from the same family, carrying a THRB gene variant, underwent detailed clinical evaluation and serum biochemistry analysis. The genetic assessment involved sequencing of the THRB gene. Hearing loss assessment included (i) meatoscopy, (ii) audiometric tests using pure tone audiometry, (iii) middle ear evaluation by tympanometry, (iv) transient otoacoustic emissions (TOAE), and (v) computed tomography of the mastoids. Genetic sequencing confirmed the THRB gene alteration (p.M442T) in three family members. All affected subjects had clinical and laboratory RTH features. Notably, the older subject with RTH was affected by a bilateral sensorineural hearing loss pattern affected by high frequencies, and cochlear dysfunction was also presented by TOAE analysis, indicating pronounced hearing loss. Hearing loss represents a significant concern in subjects with RTH, emphasizing the need for continuous and comprehensive audiological assessments. These findings underscore the importance of lifelong monitoring, particularly to assess age-related progression of hearing impairment.

## INTRODUCTION

Resistance to thyroid hormone syndrome (RTH) occurs in approximately 1 in 40,000 to 50,000 live births. In approximately 85% of cases, inheritance is autosomal dominant, with variants in the thyroid hormone receptor beta (THRB) gene being the underlying cause (^1^). A key characteristic of RTH is the elevated levels of free thyroxine (fT_4_) and triiodothyronine (fT_3_) in the bloodstream, which can range from mildly elevated to significantly above the upper limit, concomitant with normal (non-suppressed) circulating serum levels of thyroid-stimulating hormone (TSH) (^2^,^3^).

The clinical manifestations of RTH are highly diverse, encompassing a spectrum that ranges from asymptomatic cases to symptoms of hypothyroidism in tissues with a high prevalence of THRB expression, such as hearing impairment and recurrent ear infections. Alternatively, hyperthyroidism can occur in tissues where thyroid hormone receptor alpha (THRA) predominates, or in some cases, both hyperthyroidism and hypothyroidism can occur in different tissues (^3^,^4^). Despite inter-individual and interfamilial variations in the condition’s presentation, there is an evident correlation between genotype and phenotype. The varying degrees of severity can be attributed to each alteration’s specific characteristics and location (^5^).

The THRB is expressed during specific developmental stages of sensory tissues, including the retina, inner ear, and cochlea. Its expression pattern indicates the cochlea as a site of active action of thyroid hormone (TH) action. Consequently, the absence or reduction in the transport of these hormones during early developmental periods can lead to permanent morphological abnormalities in the spiral, impairing cochlear function (^6^). Studies involving THRB-knockout mice and subjects homozygous for THRB variants have demonstrated this association with severe hearing impairment (^7^,^8^,^9^).

Hearing loss occurs in approximately 20% of subjects with RTH (^10^). However, the long-term impact of inadequate TH action on the auditory system remains poorly understood, particularly regarding whether cochlear dysfunction worsens with age. Research on RTH animal models has shown disruptions of ear homeostatic function, suggesting that additional age-related hearing impairment may occur in adulthood (^11^). This study reports audiological changes in three adult family members carrying a THRB gene variant.

## SUBJECTS AND METHODS

### Clinical phenotype, serum biochemistry, and genotyping characterization

Fifteen of 82 subjects, all first- and second-generation members of the same family, carried the p.M442T variant for the THRB gene. These subjects underwent an initial evaluation involving complete thyroid hormone evaluation (TSH, fT_4_, and fT_3_). Only subjects with thyroid function abnormalities compatible with RTH initially joined the clinical protocol and molecular testing. We identified one family member carrying the p.M442T THRB gene variant. Consequently, we expanded the laboratory and genetic testing to his immediate family. The clinical team gathered detailed clinical information history about each patient using a standard physician questionnaire.

Automated techniques determined the blood count, namely fluorescent and impedance flow cytometry. When applicable, microscopy confirmed counts and morphological analysis on the Sysmex XN equipment. The enzymatic assay calculated the LDL based on Friedewald and Martin’s formulas and total cholesterol, triglycerides, and HDL. The CPK was measured using the kinetic method, while glucose was evaluated using the enzymatic method. Both analyses used COBAS C501 equipment and the ROCHE Kit. SHBG, osteocalcin, insulin, TSH, and ferritin were quantified via electrochemiluminometric method. Free T_4_ (fT_4_) and T_3_ (fT_3_), anti-thyroid peroxidase, and anti-thyroglobulin antibodies were measured by electrochemiluminescence immunoassay using COBAS E602 equipment and the Roche kit.

A Micromed device recorded the electrocardiogram. Bone densitometry and body composition were assessed using the absorption technique of two low-energy beams emitted by X-rays (DXA) on a densitometer model Lunar Prodigy Advance. Standard scanning modes of 37.0 µGy and 0.4 µGy were employed, respectively.

A Philips HD11 with a 7 MHz linear probe captured the thyroid ultrasound with color Doppler. Computed tomography of the mastoids used a Philips Brilliance 64 device, acquiring axial-plane images with subsequent coronal reformatting before and after administering iodinated contrast medium intravenously.

The genetic assessment involved DNA extraction from peripheral leukocytes, PCR amplification, sequencing, and the software analysis performed, as previously described (^12^,^13^). All participants provided written consent. The Human Research Ethics Committee of the Federal University of Bahia (CAAE no. 46298620.5.0000.5662) approved the study.

### Hearing assessment

Three RTH+ subjects, free of current symptomatic ear infections, were enrolled in the study. We performed a meatoscopy to identify possible obstructions that could impede the efficacy of the planned audiometric tests. Pure-tone audiometry determined the psychoacoustic thresholds at frequencies ranging from 0.25 to 8 kHz. The examination used a Harp model Inventis audiometer and the TDH39 model on the earphone. The Interacoustics Titan handheld tympanometry evaluated the middle ear with a probe tone of 226 Hz. The type “A” curve was adopted as the standard for comparison because it represents the typical mobility of the tympanic-ossicular system.

The contralateral stapedial reflexes were considered present at normal levels if they occurred between 70 and 100 dB above the air conduction threshold at frequencies from 0.5 to 4 kHz. The cochlear function was investigated using transient otoacoustic emissions (TOAE), employing the Interacoustics’ Titan equipment with a frequency sweep from 1 to 5 kHz, a minimum reproducibility reliability of 98%, and a non-linear click stimulus of 85 dB SPL. The reference standard for TOAE was obtaining a minimum signal-to-noise ratio level ≥ 3 dB. The electrodes were positioned by the 10-20 system, with the leads recommended for recording the electrical responses of the brain stem after cleaning the skin and ensuring an impedance of ≤ 5 kΩ.

The parameters adopted from the unfiltered click stimulus, with a duration of 100 µs, stimulation speed of 17.1 clicks/s, an intensity of 80 dB NA, a total of 2000 measurements, and alternating polarity. We repeated each set of results to ensure the reproducibility and reliability of the waves. The study utilized insertion headphones, Contronic equipment, and Evokadus software. The stimuli were presented monaurally and recorded as ipsilateral to the afferent ear.

## RESULTS

### Genotyping

Genotyping revealed a T-to-C transition of the nucleotide 1325 (c.T1325>C), resulting in methionine to threonine substitution at codon 442 (exon 10) of the THRB gene (p.M442T) in three family members.

### Phenotypic description

**Table 1** summarizes the most pertinent results.

**Table 1.** Laboratory tests and thyroid hormone action biomarkers

Tests/RTH Patients	A	B	C	Reference value
TSH (mUI/L)	2,4	2,9	2,3	0,45 a 4,5
fT_4_ (ng/dL)	3,7	3,1	3,4	0,9 a 1,7
fT_3_ (ng/dL)	0,66	0,54	0,58	0,24 a 0,37
Total Cholesterol (mg/dL)	185	270	194	<190
Triglycerides (mg/dL)	100	104	79	<150
HDL (mg/dL)	48	74	42	<40
LDL (mg/dL)	116	173	134	High (160-189)
Glucose (mg/dL)	91	93	108	70-99
Insulin (mU/L)	10	14	16	From 2 to 13, with fasting glucose below 100 mg/dL and BMI up to 25 kg/m^2^
HOMA-IR	2,2	3,2	4,2	Above 2.71 related to resistance to insulin action
CPK (U/L)	300	100	222	26-140
SHBG (nmol/L)	11	44	18	Male < 49 years-old (18-54); > 50 years-old (21-77); female < 49 years-old (32-128)
Ferritin (µg/L)	207	67	561	Male (26-446); female (15-149)
Osteocalcin (ng/mL)	32,3	28,7	19,3	11-48

TSH: thyroid-stimulating hormone; fT_4_: free thyroxine; fT_3_: free triiodothyronine; HDL: high density lipoprotein; LDL: low density lipoprotein; HOMA-IR: homeostatic model assessment for insulin resistance; BMI: body mass index; CPK: creatine phosphokinase; SHBG: sex hormone binding globulin.

**Patient A** (index case): A 22-year-old male presented alopecia, palpitations, insomnia, anxiety, childhood hyperactivity, and inability to achieve muscle mass despite engaging in intense exercise. Electrocardiogram findings included sinus arrhythmia, intraventricular conduction delay, and a heart rate of 66 bpm.

**Patient B:** A 25-year-old female reported a history of alopecia, dyspnea, sweating in hands and feet, childhood repeated ear infections followed by hearing impairment, anxiety, migraines, gastroesophageal reflux, gastritis, irregular menstrual cycles, and cramps. She had a body mass index of 26.2 kg/m^2^, classifying her as overweight, and a recorded heart rate of 76 bpm.

**Patient C:** A 65-year-old male presented hearing loss, recurrent rhinosinusitis, asthenia, polydipsia, hypertension, and a history of learning difficulty. Clinical evaluation revealed a heart rate of 99 bpm, complete left bundle branch block, osteoporosis, and a benign thyroid nodule in the left lobe.

### Hearing loss assessment of subjects with resistance to thyroid hormone syndrome

**Figure 1.**
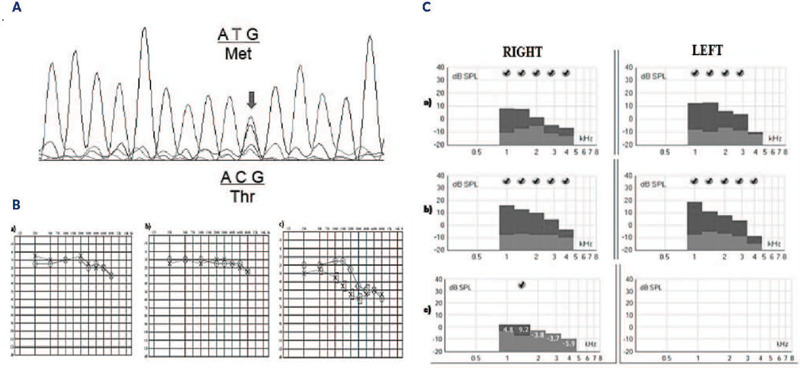
**A)** Electropherogram showing the T-to-C transition of the nucleotide 1325 (c.T1325>C) causing a methionine to threonine substitution at codon 442 (exon 10) of the TRβ gene (p.M442T) in the index case. **B)** Audiogram of patients A **(a),** B **(b)** and C **(c),** respectively. Graphical representation of hearing thresholds. On the abscissa frequencies in hertz; in the order of intensities researched until reaching the minimum audibility threshold for each frequency (from −10db, to 120 db); right ear represented by the symbol O connected by a solid line, and left ear symbol X connected by a dashed line. X and O represent air conduction, and when together with brackets represents bone conduction reaching the cochlea, they represent sensorineural hearing loss. Normality threshold up to 25 dB, if frequency above this value constitutes hearing loss. a) borderline normality. b) normal hearing. c) frequencies well above 25 db, configuring a hearing loss, predominantly in the high frequencies, characterizing a lesion in the cochlear base region. **C)** Recording of transient otoacoustic emissions from patients A **(a)**, B **(b)** and C **(c)**, respectively. Abscissa transient frequency in Hertz, in coordinates the response amplitude in dB. Analyzes the cochlear function of the right and left ear, bar represents amplitude, in gray color, at the base, represents the noise amplitude, signal-to-noise ratio (difference between the hair cell response and noise amplitude) must be greater than or equal to 6 dB (V).

A descending pattern of audiometric curves was observed, with higher frequencies slightly diminished in younger subjects (Figure 1B: a and b) and a pronounced reduction in patient C (Figure 1B: c). Figure 1C also illustrates a sensorineural hearing loss, with bilateral occurrence affecting high frequencies across different age groups. Middle ear involvement was ruled out based on acoustic impedance and the adequate mobility of the tympanic-ossicular system, which precludes the existence of conductive hearing losses.

The TOAE analysis revealed that the cochlear function, particularly the outer hair cells, exhibited enhanced response amplitudes and signal-to-noise ratios in the lower and medium frequency bands. However, these parameters declined considerably with increasing frequency in younger subjects without clearly defined degrees of hearing loss. Conversely, the older individual (patient C) lacked responses, especially in the left ear, where hearing loss was more pronounced (Figure 1C: c). Additionally, the auditory brainstem evoked potentials and revealed no signs of central auditory changes at all subjects’ VIII cranial nerve and brainstem levels.

The computed tomography examination for the three adults showed a deviation of the nasal septum, with patient C exhibiting mild diffuse cerebral involution and degenerative osteoarticular process of the temporomandibular joints.

## DISCUSSION

The RTH clinical phenotype was confirmed in patients A, B, and C through laboratory tests demonstrating elevated fT_3_ and fT_4_ with unsuppressed TSH. Genetic sequencing further corroborated this finding (^4^). A patient with RTH exhibiting atrial fibrillation reported previously this alteration in p.M442T (^14^), but its description in the literature is limited, and functional studies are lacking (^14^). Nonetheless, its location within an evolutionarily conserved region of the THRB ligand-binding domain suggests its potential significance (^15^,^16^,^17^).

The proposed pathophysiological mechanism of RHT, including the p.M442T variation identified in this study, consists of the inability or decreased sensitivity of the altered receptor to bind to thyroid hormone. This deficiency leads to decreased dissociation of co-repressors and impaired recruitment of coactivators, preventing transcriptional gene activation (^2^,^3^).

The RTH in humans is typically associated with mild auditory phenotype, and deafness is rarely observed in patients with THRB heterozygous altered. This observation suggests that dominant alteration in THRB may exert distinct effects on the auditory system (^18^). Other genetic conditions, such as Pendred syndrome, present sensorineural hearing loss that develops in late childhood with bilateral impairment. In such cases, the SLC26A4 gene is the most frequently affected (^19^,^20^).

Previous studies suggest that hearing disorders in patients with RTH can result from both direct cochlear malformation and indirect effects through ear infections (^10^). This study observed a bilateral sensorineural hearing loss pattern affecting high frequencies in the older subject with RTH. We hypothesized that, in addition to mediating TH-dependent transcriptional control during the critical period of inner ear development, TRs may also regulate transcription without TH (^21^). Although the altered THRB is insensitive to TH in the auditory system of patients with RTH is insensitive to TH, it may retain normal TH-independent functions necessary for auditory processes, which could change over their lifetime (^18^).

The study’s small sample size limits its findings, and the research design does not provide accuracy for analyzing the degenerative processes associated with hearing loss over time. However, we hypothesize that this condition may predispose subjects to early-onset presbycusis, leading to a more pronounced effect on hearing.

Both THRB isoforms (β1 and β2) are essential for normal development of the organ of Corti and the whole auditory system. TRb^-/-^ knockout mice exhibit severe hearing impairment and abnormal electrophysiologic maturation of cochlea inner hair cells (^22^,^23^,^24^). Studies using animal models for human patients carrying heterozygous THRB variants (Thrb_PV_/+) showed no auditory phenotype, while homozygous animals Thrb_PV_/Thrb_PV_ presented severe hearing loss (^8^). However, mice homozygous for THRB1 alteration (b1/b1) have mild retardation in hair cell development during youth, progressing to significant hair cell loss in older adulthood (^11^).

Interestingly, hearing loss in mice with heterozygous THRB alteration manifests at 4 months, while homozygous alterations cause early changes as soon as 3 weeks of age (^8^). This suggests that THRB1 might be less required for hair cell formation during the embryonic period; it becomes increasingly essential for hair cell survival and auditory function in adult life (^11^). This finding aligns with our study, which detected progressive cochlear impairment throughout life attributed to the older affected subject with RTH. The impairment, identified through TOAE, reflects damage to the biomechanical properties of outer hair cells in the basal region of the cochlea, the tonotopic portion responsible for encoding high frequencies.

Animal models further reveal that THRB2 deficiency leads to minimal hair cell loss at 6 months of age, emphasizing the importance of THRB1 for hair cell integrity (^11^). Additionally, in our patients with RTH, the strong influence of the THRB1 gene expression on hearing losses of cochlear origin is supported by the lack of changes in the electrical conduction of the acoustic signal in the central auditory pathways, as demonstrated by typical ABR results, which may exclude the hypothesis of retrocochlear hearing loss as the primary factor associated with hearing loss (^6^).

In conclusion, our sample exhibited slightly elevated pure-tone thresholds in young subjects with a more severe phenotype in the older affected patient. This trend aligns with previous studies on mice carrying heterozygous alterations (^11^). However, sensorineural hearing loss phenotype in RTH can also be secondary to the THRB alteration low penetrance among subjects with the same variant. Alternatively, age-specific modifiable risk factors, such as ototoxic effects from recurrent infections, environmental exposures, or a combination of these factors, could be determinants. Hearing loss is a significant concern in subjects with RTH, emphasizing the need for continuous comprehensive audiological assessments. These findings support the importance of lifelong monitoring to prevent the progression of hearing loss with age.

## Data Availability

the data sets analyzed during the current study are available in the table complementary.
